# Nicotine Increases Alcohol Intake in Adolescent Male Rats

**DOI:** 10.3389/fnbeh.2017.00025

**Published:** 2017-02-22

**Authors:** Armando Lárraga, James D. Belluzzi, Frances M. Leslie

**Affiliations:** ^1^Department of Pharmacology, University of CaliforniaIrvine, CA, USA; ^2^Department of Anatomy and Neurobiology, University of CaliforniaIrvine, CA, USA

**Keywords:** adolescence, ethanol, *kappa*-opioid receptors, norBNI, 2-bottle choice, sex differences, tobacco, nicotine, Ethyl Alcohol (PubChem CID: 702);, nicotine (PubChem CID: 89594);, norbinaltorphimine (PubChem CID: 5480230);, and U50,488H (PubChem CID: 135349).

## Abstract

**Background:** Use of alcohol and tobacco, the two most concurrently abused drugs, typically first occurs during adolescence. Yet, there have been no systematic analyses of ethanol (EtOH) and nicotine (Nic) interactions during adolescence. Recent animal studies report that *kappa*-opioid (KOR) receptor activation mediates age differences in drug reinforcement. Our hypothesis is that concurrent self-administration of EtOH and Nic will be greater in adolescent rats because of age differences in KOR function. Furthermore, exposure to alcohol and nicotine during adolescence has been reported to increase EtOH intake in adulthood. We performed a longitudinal animal study and hypothesized adolescent rats allowed to self-administer nicotine would drink more alcohol as adults.

**Methods:** Adolescent, postnatal day (P)32, and adult (P90) male and female Sprague-Dawley rats were allowed to self-administer EtOH, Nic, or a combination of both, EtOH+Nic, in an intravenous self-administration paradigm. The role of KOR was pharmacologically evaluated with the KOR antagonist, norbinaltorphamine (norBNI) and with the KOR agonist, U50,488H. Alcohol drinking was subsequently evaluated with male rats in a drinking in the dark (DID), 2-bottle choice test.

**Results:** Concurrent Nic increased EtOH intake in adolescent males, but not in adults or females. Pharmacological blockade of KOR with norBNI robustly increased EtOH+Nic self-administration in adult male rats, but had no effect with female rats. Lastly, in our longitudinal study with male rats, we found prior self-administration of Nic or EtOH+Nic during adolescence increased subsequent oral EtOH intake, whereas prior self-administration of EtOH alone in adults increased subsequent EtOH drinking.

**Conclusions:** There are major age- and sex-differences in the reinforcing effects of EtOH+Nic. Adolescent males are sensitive to the reinforcing interactions of the two drugs, whereas this effect is inhibited by KOR activation in male adults. Nicotine self-administration in adolescent males also increased subsequent oral EtOH intake. These findings suggest that brain mechanisms underlying the reinforcing effects of EtOH and nicotine are both age- and sex-dependent, and that tobacco or e-cigarette use may increase the vulnerability of teenage boys to alcohol abuse.

## Introduction

Tobacco use consistently shows a strong positive correlation with alcohol use. Over 80% of alcoholics smoke (Batel et al., 1995), and alcohol abuse is 10–14 times more common among smokers (DiFranza and Guerrera, 1990). Most people begin drinking and smoking as teenagers (Behrendt et al., 2009), and alcohol and tobacco co-use is higher among younger (18–24 years old) than older age groups (Falk et al., 2006). Those who initiate smoking at age 13 or younger are twice as likely to abuse alcohol as those who start at age 17 or above (Falk et al., 2006). Animal behavior studies have shown adolescents find nicotine more rewarding and less aversive (Belluzzi et al., 2004; Wilmouth and Spear, 2004; Shram et al., 2006; Brielmaier et al., 2007). Adolescent rats are also less sensitive to the sedative and acute withdrawal effects of alcohol than adults (Doremus et al., 2003, 2005; Varlinskaya and Spear, 2004). Therefore, it is important to study and compare alcohol and nicotine^1^ interactions in both adolescents and adults, and examine the effects on subsequent alcohol drinking behavior.

Sex differences have also been identified in both alcohol and nicotine addiction research publications. Nicotine has been shown to increase alcohol consumption and enhance arousal in men, while decreasing alcohol consumption and positive mood in women (Acheson et al., 2006). Consistent with this, women, but not men, drink less alcohol after *ad libitum* smoking (Perkins et al., 2000). Conversely, alcohol has been shown to increase smoking behavior in men, but not women (King et al., 2009). These studies suggest that alcohol and nicotine have sex-dependent interactions. Preclinical studies have also reported sex differences in nicotine- or alcohol-induced behaviors; however there have been no studies to date comparing the combination of nicotine and alcohol between sexes. Studies evaluating nicotine have reported female rats acquire self-administration of lower doses of nicotine and have higher breakpoint values at a progressive reinforcement schedule than males (Donny et al., 2000). Studies evaluating alcohol similarly report females display conditioned place preference to lower doses of alcohol than male rats (Torres et al., 2014). These studies suggest females may be more sensitive to the rewarding properties of nicotine and alcohol, respectively. Furthermore, the reported sex differences may be age-dependent, as adolescent rats do not show sex differences in aversion to alcohol (Schramm-Sapyta et al., 2014) or nicotine reward (Chen et al., 2007).

Recent studies attribute *kappa*-opioid receptor (KOR) activation as the mediator of age differences in drug reinforcement. KOR is the opioid receptor that binds and is endogenously activated by dynorphin A (Chavkin et al., 1982). KORs are widely distributed in the brain (Mansour et al., 1995) and have been shown to induce a compensatory decrease in reward state by inhibiting dopamine release in the nucleus accumbens (Zapata and Shippenberg, 2006). KOR agonists have been shown to reduce self-administration of alcohol (Nestby et al., 1999; Lindholm et al., 2001). In contrast, stress-induced KOR activation enhances nicotine reward (Lemos et al., 2012; Smith et al., 2012). Pharmacological blockade of KOR with norbinaltorphimine (norBNI), a KOR antagonist, has been reported to both increase (Mitchell et al., 2005; Anderson et al., 2012; Morales et al., 2014) and decrease (Walker and Koob, 2008; Walker et al., 2011) alcohol reward in rats. Recently, the relationship between drug reward and the *kappa*-opioid receptor (KOR) has been shown to be age-dependent for both alcohol (Anderson et al., 2014) and nicotine (Tejeda et al., 2012).

In the present study, we examined the reinforcing effects of concurrent self-administration of alcohol and nicotine in adolescent and adult, male and female rats. Our laboratory has previously shown adolescent nicotine pretreatment enhances acquisition of EtOH self-administration (Dao et al., 2011), and self-administration of a nicotine + acetaldehyde mixture is enhanced in adolescents (Belluzzi et al., 2005). Hence, our hypothesis was that adolescents would be more sensitive than adults to the reinforcing effects of combined alcohol and nicotine (EtOH+Nic), and that age differences would be more pronounced in males than females. Furthermore, we examined the role of KOR in mediating EtOH and Nic reinforcement with a KOR antagonist (norBNI) and agonist (U50-488H). Lastly, we conducted a longitudinal study to test if *self-administration* of EtOH, Nic, or EtOH+Nic during adolescence influences subsequent alcohol preference in adulthood.

## Materials and methods

### Animals

**Figure 1:**
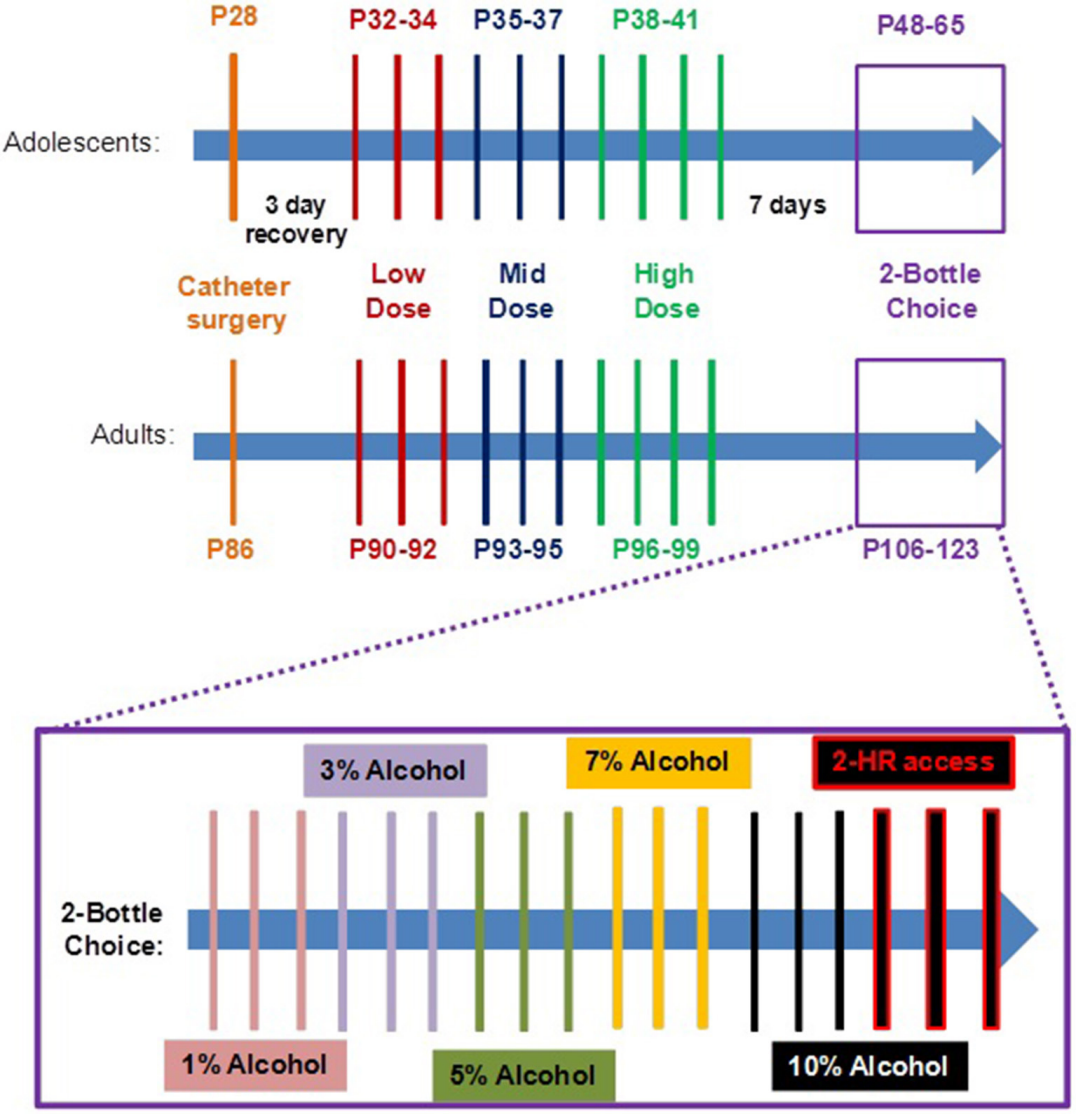
**Schematic illustration of the experimental design**. Adolescents aged postnatal day (P) 28 and adults (P86) were surgically implanted with an intravenous catheter. At P32 or P90, adolescents and adults, respectively, began intravenous self-administration (IVSA) of EtOH and nicotine (Nic), alone or in combination (EtOH+Nic) at escalating doses over 10 consecutive days. Seven days following the conclusion of IVSA, the same male rats, only, were tested in an overnight 2-bottle choice paradigm with water and increasing concentrations of unsweetened EtOH solution After escalating to a 10% EtOH solution, alcohol preference was measured during a limited 2-h access (7 p. m. to 9 p. m.); all animals had reached adulthood (Age > P60) at this time.

Male and female Sprague Dawley rats were obtained from Charles River at postnatal day (P)18 and housed with a dam until weaning (P21). Weaned juveniles and adults (P79) were group housed in an AALAC-accredited vivarium on a 12-h light-dark cycle (7 p. m. to 7 a. m.) with food and water available *ad libitum*. No more than one animal per litter was used per experimental group. All procedures were in compliance with NIH guidelines and were approved by the Institutional Animal Care and Use Committee of the University of California, Irvine. Animal suffering and the number of animals used in this study were minimized as much as possible. All animals were handled daily 3 days prior to surgery and thereafter. Consistent with our other studies (Belluzzi et al., 2005; McQuown et al., 2007; Dao et al., 2011), intravenous self-administration experiments were performed during the light cycle, whereas drinking in the dark (DID) 2-bottle choice experiments were conducted during the dark cycle. Figure 1 schematically illustrates the experimental protocol.

### Drugs

(−)-Nicotine di-(+)-tartrate (Nic) was purchased from Sigma (St. Louis, MO), 100% ethanol (EtOH) from Gold Shield distributors (Hayward, CA), norbinaltorphamine (norBNI), and U50,488H from Tocris Biosciences (Minneapolis, MN), and propofol from Abbot Laboratories (Chicago, IL). All drugs were dissolved in saline and filtered through sterile filters (Millipore Millex Sterile Filters, 0.22 μm pore, 3.3 mm diameter). Nic concentrations (pH 7.4) were calculated as free base, and EtOH was prepared at concentrations no greater than 10% EtOH (v/v).

### Catheterization

Adolescent and adult rats underwent surgery at P28 and P86, respectively. Animals were anesthetized with Equithesin (0.035 mg/kg, i.p.), and were surgically implanted with a catheter into their right jugular vein (Belluzzi et al., 2005). Rats were given 3 days to recover before beginning experiments. Cannulas were flushed daily with heparinized saline solution to maintain catheter patency. Propofol (5 mg/kg, i.v.) was injected only once, following the last self-administration session; data were discarded from animals that did not display rapid (5–10 s) anesthesia.

### Intravenous self-administration (IVSA)

At P32 and P90, respectively, adolescent and adult rats initiated intravenous self-administration (IVSA) of EtOH, Nic, EtOH+Nic, or saline in daily 2-h sessions at a fixed ratio (FR) 1-reinforcement schedule. Though intravenous self-administration has been rarely used for alcohol studies, both humans (Plawecki et al., 2013) and animals (Hyytiä et al., 1996; Dao et al., 2011) self-administer alcohol intravenously. Animals were placed in an operant chamber equipped with a house light, two nose-poke holes, and cue-lights directly above each nose-poke hole. Following each infusion there was a 3-sec time out, during which animals could not receive drug. Drug reinforcement was indicated by significant differences between responding at reinforced and non-reinforced holes. Drug doses were escalated over 10 consecutive days. We began Nic at 7.5 μg Nic/kg/infusion (Low dose), a dose that is self-administered by both adolescents and adults (Gellner et al., 2016), and maintained for the first 3 days. We then escalated to standard IVSA Nic doses: 15 μg Nic/kg/infusion (Mid dose) for days 4–6 and 30 μg Nic/kg/infusion (High dose) for days 7–10. For EtOH doses, we began with 1 mg EtOH/kg/infusion as the Low dose, and increased this dose by log scale to 10 mg EtOH/kg/infusion (Mid dose), and 100 mg EtOH/kg/infusion (High). In order to keep EtOH solutions below 10% (v/v), infusion volumes were increased as drug dose was escalated. However, infusion volumes did not differ between drug groups; animals self-administering Nic alone had the same infusion volumes as those self-administering EtOH and EtOH+Nic at each drug dose.

### Two-bottle choice

A longitudinal study with male rats was used to evaluate alcohol drinking after adolescent self-administration of EtOH, Nic, or EtOH+Nic. Alcohol drinking was evaluated on the same adolescent and adult male rats that completed the 10-day intravenous self-administration study. To allow the younger experimental rats to mature into adulthood, and allow the drugs from the intravenous self-administration (IVSA) experiments to clear out, the 2-bottle choice paradigm began 1 week after the IVSA study was complete. For 2-bottle choice experiments, male rats were single housed overnight for 12 h and allowed to drink from two bottles: one containing water, and the other EtOH. During the day, while 2-bottle choice experiments were not being conducted, all animals were group-housed in their original cages. Placement of the water and EtOH bottles was rotated each night to prevent a side preference by our animals. Rats were offered escalating EtOH concentrations over 15 consecutive nightly trials as follows: 1% EtOH (v/v) for trials 1–3, 3% EtOH (v/v) for trials 4–6, 5% EtOH (v/v) for trials 7–9, 7% EtOH (v/v) for trials 10–12, and 10% EtOH (v/v) for trials 13–15. These EtOH concentrations are standard for 2-bottle choice experiments. However, saccharine was not used in our 2-bottle choice experiments. Following the 15-trial overnight two-bottle choice procedure, rats underwent a limited access paradigm for 3 consecutive nights. At this point, all animals were over 60 days old and were considered to be adult rats. Each evening at the beginning of the dark cycle, rats were given access to a bottle of water and a bottle of 10% EtOH solution for 2 h only. After 2 h, the bottles were removed and the remaining liquid was measured to determine the amount of fluid the rats drank from each bottle.

### Role of *Kappa*-opioid receptors (KORs) in acquisition of EtOH+Nic self-administration

Separate groups of animals were prepared with jugular catheters, and treated with the irreversible *kappa*-opioid receptor antagonist, norBNI (0, or 10 mg/kg, i.p.), 1 day after surgery. A single dose of norBNI at 10 mg/kg inhibits activation of KOR for more than 21 days *in vivo*, and is dependent on c-Jun N-terminal kinase 1 activation (Melief et al., 2011). Hence, a dose response with norBNI is not necessary. Three days following norBNI or saline pretreatment, at P32 and P90, respectively, adolescent and adult rats were allowed to intravenously self-administer EtOH, Nic, or EtOH+Nic at the Low dose for three consecutive days. To evaluate the effect of direct KOR activation on EtOH+Nic self-administration, a separate groups of adolescent males was catheterized and allowed to self-administer EtOH+Nic for three consecutive days. On the fourth day, the specific KOR agonist, U50,488H (0, 0.3, 1.0, or 3.0 mg/kg, i.v.) was administered immediately before beginning the final EtOH+Nic self-administration session.

### Data analysis

Mean reinforced and non-reinforced responses for the last 2 days of intravenous self-administration of each drug dose were analyzed with a 4-way ANOVA for Age x Drug x Response (Reinforced/Non-Reinforced) x Dose (Low, Mid, and High), with repeated measures on Response and Dose. Data from animals pretreated with norBNI were analyzed by a 4-way ANOVA for Age X Pretreatment X Drug X Response, with repeated measures on Response. Results from the U50,488 experiment were also analyzed by a 2-way ANOVA for Dose X Reinforcement. Total EtOH and Nic intake during intravenous self-administration in male or female rats were analyzed separately by 3-way ANOVA for Age x Drug x Dose. Significant differences were further analyzed with ANOVAs and *post-hoc* Bonferroni-corrected *t*-tests, where appropriate.

For 2-bottle choice data, mean oral alcohol consumption for the three limited access trials was calculated by dividing alcohol intake by total fluid intake (alcohol + water). Mean alcohol consumption was analyzed with a 2-way ANOVA for Drug (prior exposure) X Age. Significant differences within each age were further analyzed with one-way ANOVA and *post-hoc* Dunnett or Bonferroni tests. Age differences were analyzed by Bonferroni-corrected unpaired *t*-tests.

Data from norBNI-treated male and female rats were analyzed separately. Mean reinforced and non-reinforced responses for the last 2 days of intravenous self-administration of each drug group were analyzed with a 3-way ANOVA for Age x Pretreatment x Reinforcement (Reinforced/Non-Reinforced Responses) with repeated measures on Reinforcement. Significant differences were further analyzed with ANOVAs and *post-hoc* Bonferroni-corrected *t*-tests. Significant effects of Reinforcement were analyzed by Bonferroni-corrected paired *t*-tests between Reinforced and Non-Reinforced Responses for each treatment group.

## Results

### Combining EtOH and Nic is reinforcing in adolescent, but not in adult males

**Figure 2:**
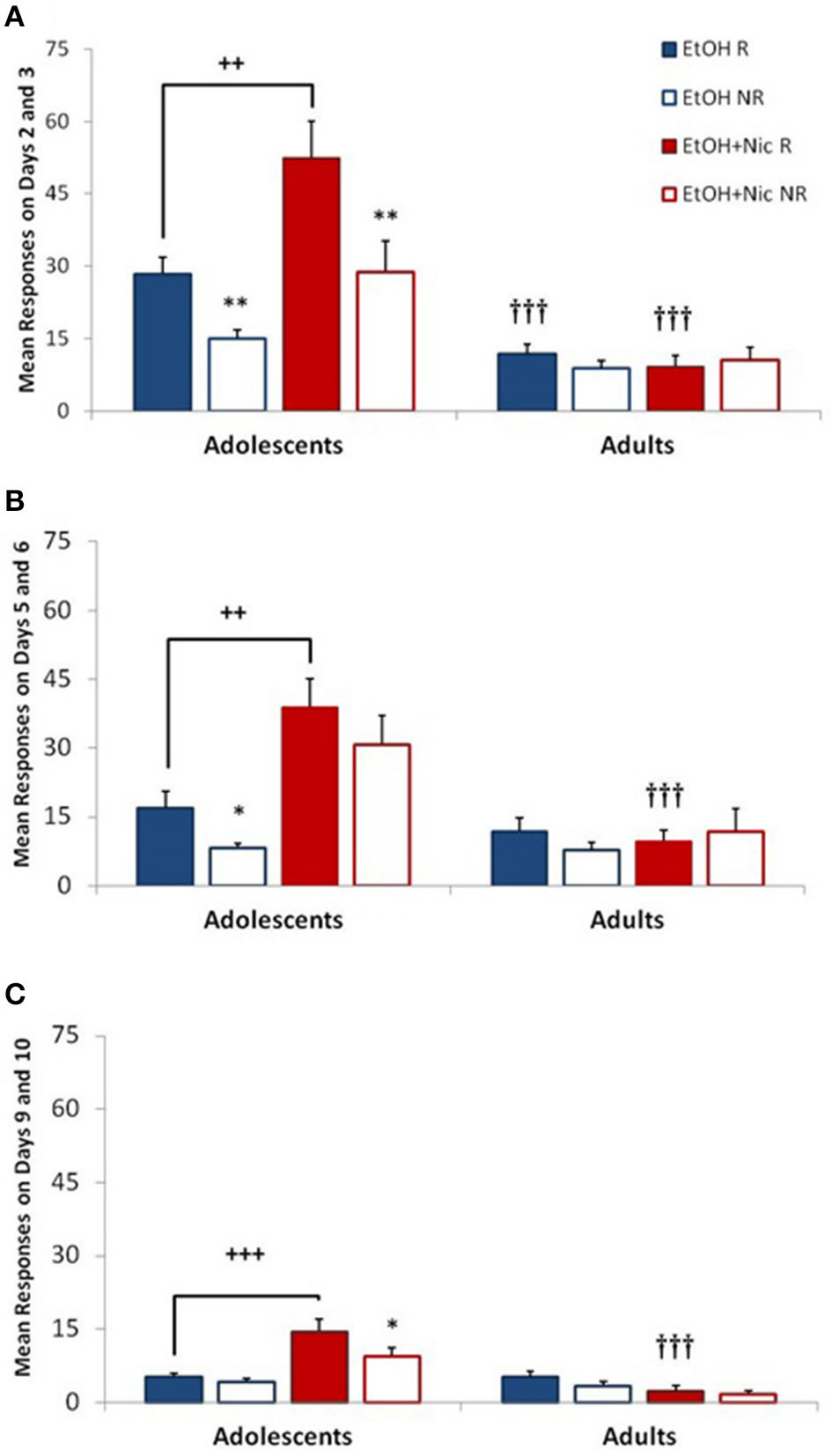
**Combining EtOH and Nicotine is reinforcing in adolescent, but not adult males**. Adolescent and adult rats self-administered EtOH alone, or in combination with Nic (EtOH+Nic) at **(A)** a Low dose (7.5 μg Nic, 1 mg EtOH/kg/infusion), **(B)** a Mid dose (15 μg Nic, 10 mg EtOH/kg/infusion), and **(C)** a High dose (30 μg Nic, 100 mg EtOH/kg/infusion). A reinforcing effect of drug is indicated by significantly higher reinforced (“R,” closed bars) than nonreinforced (“NR,” open bars) responses (^**^, *p* < 0.01; ^*^, *p* < 0.05). Reinforced responses (R) for EtOH+Nic were significantly higher than for EtOH alone in adolescents (+++, *p* < 0.001; ++, *p* < 0.01), but not adults. Reinforced responses in adolescents were significantly higher than adults (^†††^, *p* < 0.001) for EtOH at the Low dose, and for EtOH+Nic at all three test doses. Data represent mean + SEM responses averaged over the last two days at a given drug dose, *n* = 9–16 males per group.

Our initial self-administration experiment was done with adolescent and adult male rats. We observed significant age differences in the acquisition of self-administration of EtOH, and the combination of EtOH+Nic (Figure 2). Since overall ANOVA indicated significant Dose interactions with Age [*F*_(2, 98)_ = 12.383, *p* < 0.001], and Response [*F*_(2, 98)_ = 4.256, *p* = 0.017], responses at each Dose were analyzed separately.

At the Low dose (Figure 2A), there was an overall Age X Drug interaction [*F*_(1, 49)_ = 5.858, *p* = 0.019]. Adolescent males found EtOH, alone and in combination with Nic (EtOH+Nic), to be reinforcing, as indicated by significant differences between reinforced and non-reinforced responses (^**^, *p* < 0.01). Adolescents also self-administered significantly more EtOH+Nic than EtOH alone (++, *p* = 0.009). In contrast, adult male rats did not find either EtOH or EtOH+Nic reinforcing, and self-administered significantly less EtOH and EtOH+Nic than adolescents (^†††^, *p* < 0.001).

At the Mid doses (Figure 2B), there was a significant Age X Drug interaction [*F*_(1, 49)_ = 9.149, *p* = 0.004]. EtOH continued to be reinforcing in adolescent male rats at this higher dose, as indicated by significant differences between reinforced and non-reinforced responses (^**^, *p* = 0.01). The combination of EtOH+Nic, however, was not reinforcing at the Mid dose. Yet, adolescent rats self-administered larger amounts of the drug combination than EtOH alone; reinforced responses for EtOH+Nic were significantly higher reinforced responses for EtOH in adolescents (++, *p* = 0.002) or for EtOH+Nic in adults (^†††^, *p* = 0.001). Adult male rats did not show a reinforcing effect to EtOH or EtOH+Nic at the Mid dose.

At the High dose (Figure 2C), there was a significant Age X Drug interaction [*F*_(1, 49)_ = 13.274, *p* = 0.001], and significant effect of Responses [*F*_(1, 49)_ = 11.365, *p* = 0.001]. The only drug treatment that was reinforcing was EtOH+Nic in adolescents (^*^, *p* < 0.05). Adolescent males also self-administered significantly more EtOH+Nic than EtOH (+++, *p* = 0.001). Lastly, reinforced responses for EtOH+Nic were also significantly higher in adolescent than adult males (^†††^, *p* = 0.001).

### Combining alcohol and nicotine increased drug intake in adolescent male rats

**Figure 3:**
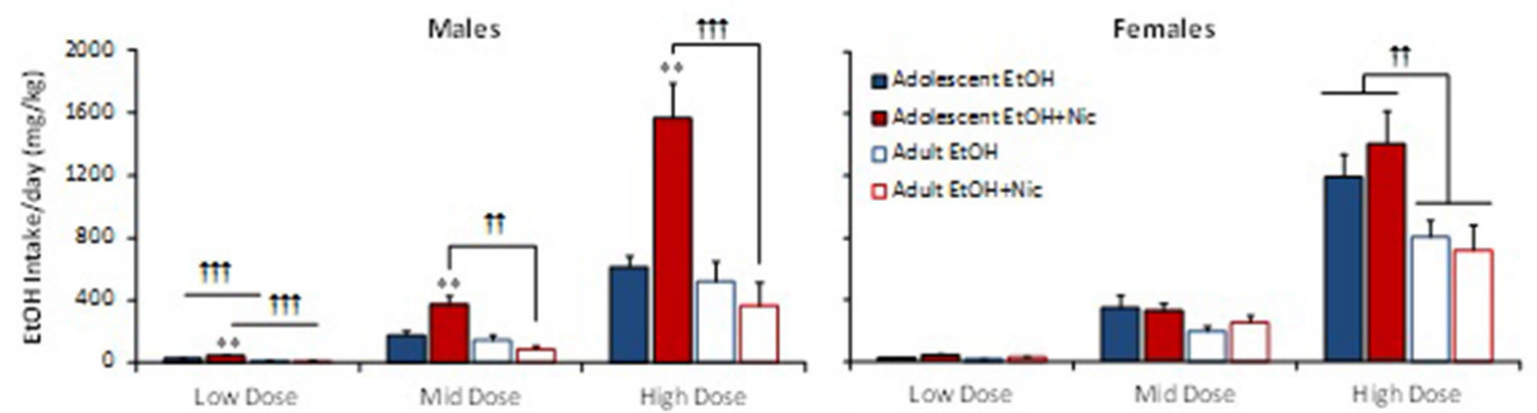
**Combining alcohol and nicotine increases EtOH intake in adolescent males, but not adult males or females**. EtOH intake of male (left panel) and female (right panel), adolescent (filled bars) and adult (open bars), rats at Low, Mid and High test doses. Combining EtOH and Nic (EtOH+Nic) significantly increased EtOH intake compared to EtOH alone in male adolescent rats (^**^, *p* < 0.01,) but not in male adult or female rats. EtOH intake is higher in male adolescents than male adults at the Low dose (^†††^, *p* < 0.001), and at the Mid and High EtOH+Nic doses (^††^, *p* < 0.01). Female adolescents had a higher EtOH intake at the High dose than female adults (^††^, *p* < 0.01). Data represent mean + SEM of the last 2 days of intravenous self-administration at each test dose for each group, *n* = 9–16/group.

Since we observed a significant age difference in EtOH+Nic self-administration that persisted over three different dose combinations, we also included female adolescent and adult rats, and compared drug intake between age and sex. Nic significantly increased EtOH intake in adolescent, but not adult males (Figure 3). EtOH intake in males showed a significant Dose x Age x Drug interaction [*F*_(2, 96)_ = 10.652, *p* < 0.001]; hence, each dose was analyzed separately. At all three test doses, the adolescent EtOH+Nic group had significantly higher EtOH intake than the adolescent EtOH alone group (^**^, *p* < 0.01). In addition, adolescent males self-administered more EtOH than adults at the Low, Mid, and High doses of EtOH+Nic, and at the Low dose of EtOH alone (^†††^, *p* < 0.001; ^††^, *p* < 0.01 vs. adults). In contrast, combining Nic with EtOH did not increase EtOH intake in females. Three-way ANOVA analysis of Age x Drug x Dose was not statistically significant [*F*_(2, 78)_ = 1.143, *p* = 0.324] and did not show a statistically significant Drug effect (*p* = 0.622) or Age x Drug interaction (*p* = 0.448). Females only showed a significant Age difference at the High dose that was not dependent on Drug (^††^, *p* < 0.01).

### Age differences in KOR function mediate age-dependent effects of EtOH+Nic reinforcement

**Figure 4:**
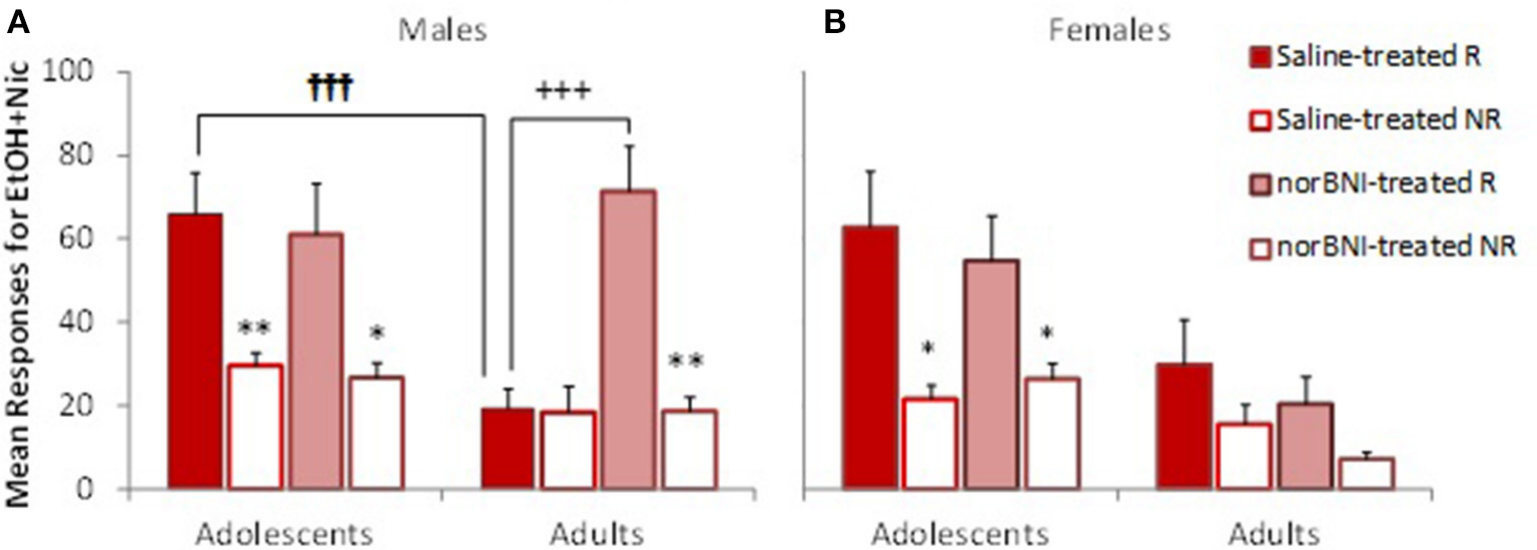
**KOR blockade increases EtOH+Nic reinforcement in adult male rats, but not adolescent males or females. (A)** Blockade of kappa-opioid receptor (KOR) with norBNI significantly increased EtOH+Nic reinforcement in adult males; reinforced (R) responses were significantly higher than non-reinforced (NR) (^**^, *p* < 0.001) and saline-pretreated reinforced responses (^+++^, *p* < 0.001). Saline-treated adolescents males had significantly higher reinforced responses than saline-treated adult males (^†††^, *p* < 0.001). **(B)** Male and female adolescent rats find EtOH+Nic reinforcing regardless of pretreatment; Reinforced (R) responses were significantly higher than non-reinforced (NR; ^**^, *p* < 0.01; ^*^, *p* < 0.05). No effect of norBNI was observed in females, *n* = 7–13/group.

To test if age differences in EtOH+Nic self-administration reflect age-dependent KOR activation, we pretreated adolescent and adult, male and female rats, with the standard dose of the irreversible KOR antagonist, norBNI or saline, and compared self-administration of EtOH+Nic at the Low dose (Figure 4). Significant overall interactions of Response x Age x Drug x Pretreatment were found for males [*F*_(2, 130)_ = 5.651, *p* = 0.004]. NorBNI pretreatment had a robust effect on EtOH+Nic reinforcement in adult male rats. Males (Figure 4A; left panel) showed a significant overall Response X Age X Pretreatment interaction [*F*_(1, 46)_ = 6.902, *p* = 0.012]. EtOH+Nic was reinforcing in norBNI-pretreated adult males: reinforced responses were significantly higher than non-reinforced responses (^**^, *p* < 0.01), and higher than reinforced responses of saline-pretreated adults (+++, *p* < 0.001). In adolescent males, norBNI pretreatment did not have an effect on EtOH+Nic reinforcement. Consistent with our first experiment, adolescent males found EtOH+Nic reinforcing regardless of pretreatment (^**^, *p* < 0.01; ^*^, *p* < 0.05). NorBNI pretreatment of male rats increased adult reinforced responses for EtOH+Nic to levels seen with adolescents (Figure 4A). In contrast, females showed no effect to norBNI pretreatment (Figure 4B; right panel). There was a significant overall Response x Age interaction [*F*_(1, 30)_ = 5.542, *p* = 0.016]; EtOH+Nic was reinforcing in adolescent (^*^, *p* < 0.05), but not adult females, regardless of pretreatment.

**Figure 5:**
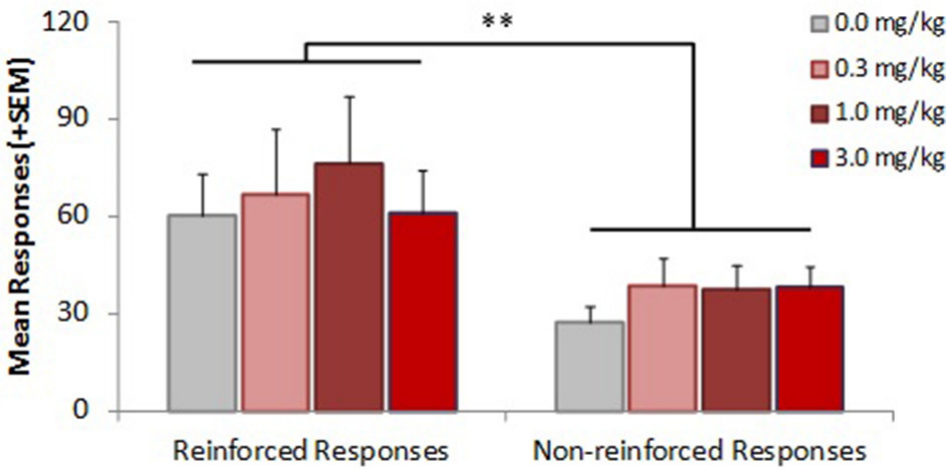
**Activating KOR does not inhibit EtOH+Nic reinforcement in male adolescent rats**. Adolescent male rats self-administering EtOH+Nic were treated with U50,488 (0, 0.3, 1.0, or 3.0 mg/kg, i.v.) on test day. No statistically significant effect of U50,488 was observed. Overall, reinforced responses were significantly higher than non-reinforced responses (^**^, *p* < 0.01), *n* = 11–13 adolescent males.

NorBNI pretreatment revealed that KOR activation inhibits EtOH+Nic reinforcement in male adults, but not adolescent males. In order to test whether KOR was functionally active in adolescent males that show a reinforcement effect to the combination EtOH+Nic, we examined the effect of the KOR agonist, U50,488, on a separate group of adolescent male rats (Figure 5). Only an overall reinforcement effect was observed [*F*_(1, 43)_ = 18.327, *p* < 0.001], with no significant inhibition of responding at any U50,488 dose.

### Nicotine self-administration during adolescence increases subsequent EtOH drinking in adulthood

**Figure 6:**
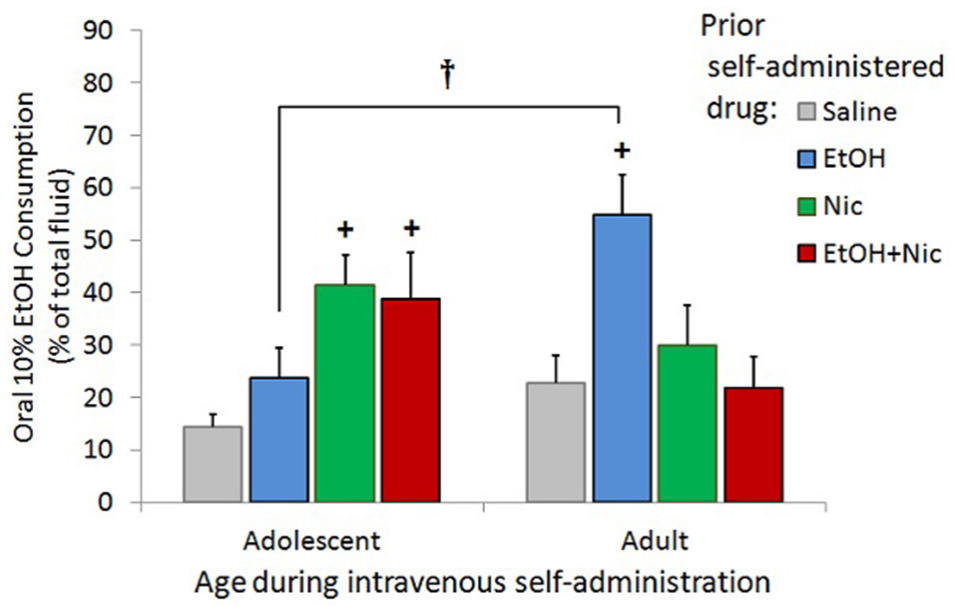
**Nic self-administration during adolescence increases subsequent alcohol drinking in adulthood**. Data are average 10% EtOH consumption as a percent of total fluid consumption over 3 consecutive trials in a limited 2-hr access 2-bottle choice paradigm with male rats that had previously self-administered saline, Nic, EtOH, or EtOH+Nic as either adolescents or adults. Rats that self-administered Nic or EtOH+Nic as adolescents drank more 10% EtOH solution than saline-control rats (+, *p* < 0.05). Rats that self-administered EtOH as adults drank more 10% EtOH solution than adult saline controls (+, *p* < 0.05) and rats that previously self-administered EtOH as adolescents (^†^, *p* < 0.05). 10% EtOH consumed as a % of total fluid is shown as mean + SEM for each treatment group, *n* = 8–13 males/group.

In order to test the effect of adolescent Nic and/or EtOH use on EtOH drinking during adulthood, we performed a 2-bottle choice experiment on the same male rats from our initial self-administration experiment. No female rats were tested in our 2-bottle choice paradigm. We found that self-administration of Nic during adolescence increased subsequent oral intake of EtOH in a drinking-in-the-dark paradigm during adulthood (Figure 6). Though none of the test groups demonstrated a preference for EtOH over water, a significant Drug x Age (during IVSA) interaction [*F*_(3, 74)_ = 5.051, *p* = 0.003] was seen for EtOH intake as a percent of total liquid consumption. Male rats that, as adolescents, self-administered Nic alone or in combination with EtOH (EtOH+Nic), drank more EtOH than saline controls (+, *p* < 0.05). Adolescent rats that intravenously self-administered EtOHalone did not subsequently drink more EtOH than saline-control animals. In contrast, rats that intravenously self-administered EtOH alone as adults did drink more EtOH than saline-controls (+, *p* < 0.05). They also drank more EtOH than their adolescent counterparts (^††^, *p* < 0.01). Notably, adults that self-administered EtOH+Nic did not increase subsequent oral EtOH intake (Figure 6), even though Nic did not significantly alter intravenous EtOH intake in this group (Figure 2A). Thus, Nic self-administration in adolescents increases subsequent alcohol drinking, whereas it inhibits the enhancing effects of EtOH self-administration on subsequent alcohol drinking in adults.

## Discussion

We have shown major age and sex differences in the behavioral interactions of nicotine and alcohol with a novel self-administration paradigm. Consistent with our earlier study in which nicotine was given as a passive pretreatment (Dao et al., 2011), we now show that concurrent nicotine self-administration enhances alcohol reinforcement and intake in adolescent male, but not adult male or female rats of either age. In contrast, concurrent self-administration of EtOH and Nic in adults activates KOR, which blocks drug reinforcement. With the use of a longitudinal study, we also show that nicotine self-administration during adolescence increases subsequent oral consumption of alcohol. These findings suggest that tobacco use by male teenagers may pose a significant risk factor for subsequent alcohol abuse. The present findings also contribute to a growing literature of substantial interactions between nicotine and alcohol, at both the molecular and circuit level (Doyon et al., 2013; Hendrickson et al., 2013).

### Nicotine increases alcohol intake when combined in adolescent males

This is the first study to examine concurrent self-administration of nicotine and EtOH in adolescents. Our intravenous self-administration paradigm was designed to avoid confounds resulting from age differences in response to alcohol taste, and to allow for a direct comparison of nicotine and alcohol reinforcement when the two are combined. Although intravenous self-administration is the standard method used in animal models of addiction, it has been rarely used for alcohol studies. However, both humans (Plawecki et al., 2013) and animals (Hyytiä et al., 1996; Dao et al., 2011) self-administer alcohol intravenously. Lastly, we used nose-pokes, not levers, in our self-administration operant chambers to facilitate spontaneous acquisition of responding with adolescent animals. Although non-reinforced activity is higher with nose pokes than levers (Clemens et al., 2010), we observed immediate acquisition of self-administration in all adolescent groups, as defined by significantly higher reinforced than non-reinforced responses.

We found significant age differences in drug reinforcement and intake with our self-administration experiments. Nic *increased* EtOH reinforcement and intake in *adolescent* males. This finding suggests adolescents have increased sensitivity to alcohol reward, as has been reported previously (Maldonado et al., 2008; García-Burgos et al., 2009); and is consistent with a study where cigarette smoke exposure increased alcohol consumption in adolescent mice (Burns and Proctor, 2013). Our study is also supported by the clinical finding that alcohol and nicotine co-use is higher among younger (18–24) than older age groups (25–44, 25–64, and 65+) (Falk et al., 2006).

Contrary to the males, EtOH intake did not differ by age in females, and was not affected by combining Nic with EtOH. Other studies have also observed sex differences in responses to nicotine and alcohol. A clinical study reported mecamylamine, a nicotinic acetylcholine receptor (nAChR) antagonist, is more effective in attenuating the positive effects of alcohol in men than in women (Chi and de Wit, 2003). Another group later showed that nicotine increased alcohol consumption in men, but decreased it in women (Acheson et al., 2006). This study also reported that nicotine enhances arousal state in men, but decreases positive mood in women. This suggests that nicotine may alter the motivation to drink differently across sexes. Consistent with this, women have been reported to drink less alcohol after *ad libitum* smoking than males (Perkins et al., 2000). These studies suggest women may not co-use alcohol and tobacco as much as men. Since our findings are consistent with rodent and human literature, we believe our self-administration model is a valid novel model to study EtOH+Nic reinforcement and the first to examine concurrent self-administration of nicotine and EtOH in adolescents.

### EtOH and Nic age- and sex-dependently interact with *Kappa*-opioid receptor function

Age differences in EtOH+Nic reinforcement in male rats were due to differences in KOR activation. Pharmacological blockade of KORs with an irreversible antagonist, norBNI, did not significantly affect responding for EtOH+Nic in females or adolescent males. However, the same treatment induced a very robust increase in EtOH+Nic reinforcement in adult males. This suggests the drug combination of EtOH and Nic induces KOR activation in adults, but not adolescents. Age differences in EtOH+Nic-induced activation of KOR may either reflect functional differences in the receptor, or in release of dynorphin, the endogenous KOR ligand (Chavkin, 2013). In order to evaluate the underlying KOR mechanism, we treated adolescent male rats with the KOR agonist, U50,488H, immediately before beginning an intravenous EtOH+Nic self-administration session. The fact that U50,488H did not inhibit EtOH+Nic reinforcement in adolescent males, suggests that the observed age difference in KOR function reflects an alteration in receptor function rather than an age difference in dynorphin release. This finding complements the conclusions of Tejeda et al. (2012), who showed that chronic nicotine increases KOR function in adults, but not adolescents. However, norBNI has recently been shown to inhibit dynorphin-stimulated G-protein signaling in the absence of KOR (Zhou et al., 2015). Thus, other potential mechanisms involving dynorphin systems should also be considered.

### Adolescent nicotine self-administration causes a lasting increase in alcohol preference

Following the initial intravenous self-administration experiments, the same male rats were allowed to mature and reach adulthood. Then, we allowed them to drink alcohol using a drinking in the dark paradigm (Rhodes et al., 2005; Kamdar et al., 2007). Nicotine self-administration during adolescence was found to increase subsequent EtOH consumption in adulthood. This is the first study to compare the effect of age of onset of Nic and EtOH self-administration on subsequent EtOH drinking later in life. These findings support the “gateway hypothesis” that adolescent Nic use subsequently increases drug reward; specifically, enhancing the vulnerability to develop alcohol abuse problems later in life. This long-lasting effect of nicotine on alcohol reward is consistent with the findings of a prior study that reported exacerbated ethanol withdrawal during adulthood in rats exposed to nicotine during adolescence (Riley et al., 2010), but is not consistent with another study that reported periadolescent nicotine treatment does not affect subsequent oral EtOH consumption (Smith et al., 2012). Such differences may reflect methodological distinctions, such as route and duration of nicotine administration, and the impact of choice on oral EtOH consumption. Our finding that adolescent EtOH intake did not affect subsequent oral alcohol preference also differs from other studies that passively administered EtOH, forced EtOH consumption (Pascual et al., 2009; Sherrill et al., 2011), or used EtOH preferring rats (Rodd-Henricks et al., 2002). However, our data are consistent with one study that allowed adolescent rats to voluntarily self-administer EtOH (Vendruscolo et al., 2010). This suggests that voluntary access and forced exposure to EtOH may differentially impact subsequent EtOH reward later in life; voluntary access has no effect, while forced exposure increases reward. Considering that stress has been shown to increase EtOH reward (Matsuzawa et al., 1998), it is possible the stress induced during forced EtOH exposure is the reason for the subsequent increase of EtOH reward in those other studies, not adolescent EtOH exposure itself. Consistent with this hypothesis, prior work has shown that adolescent mice show a long-term increase in EtOH preference after stress exposure (Siegmund et al., 2005). Thus, the stress involved in forced EtOH exposure may impact its effect on developing reward circuitry.

Lastly, we found adults that previously self-administered EtOH drank significantly more EtOH than saline-control animals, a finding consistent with much earlier reports (Roberts et al., 1996, 2000). Interestingly, combining nicotine during intravenous self-administration experiments eliminated the enhancing effect of EtOH. In contrast to adolescents, self-administration of Nic alone in adults did not change subsequent EtOH consumption. EtOH+Nic was not reinforcing in adult males. Hence, our findings suggest that, in adulthood, concurrent Nic inhibits the immediate and subsequent reinforcing properties of EtOH. Our findings are consistent with an epidemiological study which reported that heavier drinking in teenagers is correlated with smoking and the male gender (Poikolainen et al., 2001).

## Conclusions

Our study revealed age-and sex-dependent interactions of EtOH and Nic in mediating drug reinforcement behavior. Recent mouse studies have indicated that EtOH and Nic interact at α4- and α6-containing nicotinic acetylcholine receptors (nAChR) to mediate reward (Liu et al., 2013a,b). However, our present findings of age- and sex-dependent interactions of EtOH and Nic in mediating drug reinforcement, suggest that a more complex model may apply. Our research supports the concept that adolescents are less sensitive to KOR activation than adults (Natividad et al., 2010; Tejeda et al., 2012; Anderson et al., 2014; Morales et al., 2014). Furthermore, this is the first study to report an interaction by EtOH and Nic on KOR function, and offers an explanation to why most tobacco-using teenagers also drink alcohol (Orlando et al., 2005). The reported increase in alcohol intake when combined with Nic in male rats is a remarkable finding considering that these animals are voluntarily consuming EtOH. Our findings are supported by clinical studies reporting increases in smoking behavior with alcohol (Friedman et al., 1991; Rose et al., 2004; King et al., 2009; McKee et al., 2010), and conversely, increased alcohol drinking with nicotine (Barrett et al., 2006). Female rats exhibited vastly different behavioral responses to the drug combination than males. The self-administration findings are consistent with those of a clinical study reporting that nicotine increased alcohol consumption in men, but decreased it in women (Acheson et al., 2006). These findings suggest sex-dependent interactions of EtOH and Nic with KOR function.

Our longitudinal analysis of EtOH consumption with a drinking in the dark 2-bottle choice showed significant age of onset of drug use, and drug group differences. We found adolescent use of Nic, but not EtOH, significantly increased subsequent EtOH drinking in adulthood. This supports the “gateway hypothesis” for Nic and suggests that EtOH may not be a “gateway” drug in adolescence. Our findings are supported by an epidemiological study reporting heavier drinking in teenagers is correlated with smoking and the male gender (Poikolainen et al., 2001).

In summary, our study supports the growing body of literature suggesting age- and sex-dependent KOR function. In addition, it illustrates that EtOH and Nic interact with KOR to induce age- and sex-dependent behaviors. Alcohol research and tobacco research have always been considered separate fields. Our work incorporates the two, and suggests they are interconnected as so much clinical data suggest. For years, we have known people begin drinking and smoking as teenagers. These findings propose a biological factor for this highly co-morbid phenomenon; male adolescents are less sensitive to KOR inhibition of reinforcement induced by combining alcohol and nicotine. Our research suggests KOR activation as a novel mechanism mediating age differences in alcohol and tobacco co-abuse, and provides strong evidence that sex and age of first exposure are important determinants of the interactive effects of nicotine and alcohol. Two subtypes of alcoholism have been widely recognized: Type-A, or late onset, alcoholism develops after age 25; and Type-B, or early onset, alcoholism develops before age 25 (Johnson, 2010). Pharmacological treatment for alcoholism depends on severity of use and on age of onset. People who consistently smoke and drink as teenagers, and throughout their early twenties, experience substantially more negative consequences by age 29 than those who occasionally smoke and drink, or drink and not smoke (Orlando et al., 2005). With the recent escalating use of e-cigarettes in school-age children (Arrazola et al., 2015), concern has been raised that these may also increase the risk for alcohol abuse (Hughes et al., 2015; Kristjansson and Sigfudottir, 2015). Our present findings suggest that adolescent nicotine exposure, through use of either conventional tobacco products or e-cigarettes, may not only increase the immediate rewarding effects of alcohol in males, but may also increase long-term susceptibility to alcohol use. Such findings provide further strong arguments for limiting adolescent access to nicotine and tobacco products.

## Author contributions

All authors are responsible for the experimental design; AL created the figures, conducted the experiments, literature research, data analysis, wrote the manuscript, and obtained funding; JB consulted on statistical analysis; FL consulted on statistical analysis, wrote the manuscript, is the principal investigator, and the principal recipient for funding for this work. All authors critically reviewed content and approved final version for publication.

## Funding

This works was supported by Tobacco-related disease research program (TRDRP# 18XT-0085); the National Institutes of Health (DA 040440); and Faculty Mentor Program Fellowship and President's Dissertation Year Fellowship from the University of California, Irvine.

### Conflict of interest statement

The authors declare that the research was conducted in the absence of any commercial or financial relationships that could be construed as a potential conflict of interest.

## Footnotes

^1^Abbreviations: Ethanol (EtOH); nicotine (Nic); *kappa*-opioid receptor (KOR); norbinaltorphimine (norBNI).
